# Radiation Modifies Let-7 miRNA Binding to AGO2 Independent of Changes in Transcription to Influence Tumor Cell Radiosensitivity

**DOI:** 10.3390/ijms26178483

**Published:** 2025-09-01

**Authors:** Taqveema Ali, Charlotte Degorre, Philip J. Tofilon

**Affiliations:** Radiation Oncology Branch, National Cancer Institute, 10 Center Drive-MSC 1002, Building 10, B3B69B, Bethesda, MD 20892, USA; charlotte.degorre@nih.gov (C.D.); philip.tofilon@nih.gov (P.J.T.)

**Keywords:** Let-7 miRNAs, miRNA-AGO2, transcription, radiosensitivity, cancer, DNA damage

## Abstract

Alterations in gene expression induced by ionizing radiation (IR) were commonly explained by transcriptional activation. However, the weak correlation between mRNA and protein levels following IR indicates the significant role for post-transcriptional regulation. microRNAs (miRNAs) bound to AGO2 play a significant role in post-transcriptional regulation; however, their role in radiation response is not clear. miRNA sequencing was performed to analyze the miRNAome of glioma cells. The effect of IR on Let-7 miRNAs and their association with AGO2 was examined using RT-qPCR and RNA immunoprecipitation (RIP) assays. Clonogenic assays were performed to measure radiosensitivity following Let-7a overexpression or knockdown. DNA damage (γH2AX foci) and cell cycle distribution were analyzed by immunofluorescence and flow cytometry. Let-7 miRNA regulatory networks were identified through target prediction and pathway enrichment analysis. AGO2-Let-7 binding decreased post IR, indicating impaired RISC loading. Let-7 overexpression increased radiosensitivity, DNA damage and G2/M cell cycle arrest in glioma and other cells (HeLa and MDA-MB-231). Let-7 miRNAs mainly targeted cell cycle and DNA damage response (DDR) pathways. Our study showed radiation impairs AGO2-miRNA binding, while restoring Let-7-AGO2 interaction enhances radiosensitivity by modulating DNA repair and cell cycle checkpoint activation. Targeting AGO2-miRNA dynamics represents a promising approach to improve radiotherapy outcomes.

## 1. Introduction

Approximately 50% of patients with solid tumors receive radiotherapy during their treatment [1]. Aiming towards improving the efficacy of radiation, the standard approach involves the development and application of molecularly targeted agents, a strategy that requires a thorough understanding of the fundamental processes comprising cellular radiation response. Such processes include not only constitutively expressed proteins but also radiation-induced changes in gene expression. Along these lines, a critical regulator of gene expression is miRNA, which promotes the translational repression or degradation of client mRNAs [2,3]. Constitutively expressed miRNAs have been reported to influence cellular radiosensitivity through the maintenance of pre-existing pro-survival pathways [4,5]. In addition, microarray or RNAseq analyses of the cellular transcriptome after irradiation have identified changes in the level of specific miRNAs, which have been assumed to play a role in mediating radiation-induced gene expression [6]. However, many such changes occur at 24 h or longer after irradiation with few, if any, before or at 6 h [7,8], a time course suggesting that the reported changes in miRNA levels are primarily a consequence rather than a cause of radiation-induced gene expression. The putative role of miRNAs in mediating radiation-induced gene expression thus remains to be determined.

Investigations of radiation-induced modifications in miRNA levels have, to date, focused solely on transcriptome analysis. However, miRNA is active only after binding to AGO2 protein, which then serves as the targeting module of the miRNA-induced silencing complex (miRISC). AGO2 loaded with miRNA inhibits the expression of client mRNAs through miRNA–target base pairing [9]. Importantly, defining the expression of miRNAs in the total miRNA pool (i.e., the transcriptome) is not an accurate measure of miRNAs bound to AGO2, and consequently, it is not an accurate indicator of miRNA inhibitory potential [10]. To more thoroughly investigate the role of miRNA in regulating radiation-induced gene expression, it is necessary to determine whether radiation modifies miRNA-AGO2 binding.

To this end, we focused on the Let-7 family of miRNAs, which are among the most abundantly expressed miRNAs. There are 10 unique human Let-7 microRNAs [11]; because they share the same seed sequence, there is significant overlap in their target mRNAs [12]. The Let-7 family regulates a variety of biological processes, including proliferation and differentiation, and has been shown to act as a tumor suppressor [13,14,15]. To better understand the role of Let-7 in radiation response, we determined the effects of radiation on Let-7 expression and its binding to AGO2. The data presented show that radiation-induced little to no change in Let-7 expression yet rapidly reduced Let-7 binding to AGO2. Whereas knockdown of Let-7 had no effect on radiation-induced cell death, overexpression of Let-7, which prevented the radiation-induced decrease in Let-7-AGO2 binding, significantly increased the tumor cell radiosensitivity. Overexpression of Let-7 in normal cell lines also prevented the radiation-induced decrease in Let-7-AGO2 binding yet had no effect on their radiosensitivity. These results indicate that radiation reduces the levels of functional Let-7 miRNAs and suggest that Let-7 overexpression may provide a strategy for differentially enhancing tumor cell radiosensitivity over normal cells.

## 2. Results

### 2.1. Effects of Radiation on Let-7 miRNA Expression and AGO2 Binding in U-251 Glioma Cells

**Figure 1:**
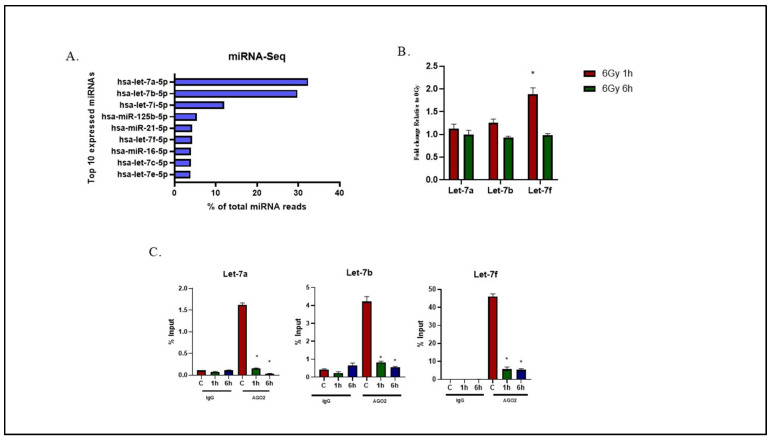
(**A**) Top 10 most abundantly expressed miRNAs in the sequencing study. The read count indicates a total unnormalized count of reads for each miRNA detected by Illumina miRNA sequencing. (**B**) qRT-PCR of Let-7a, Let-7b, and Let-7f was performed. U-251 cells were exposed to 6 Gy of radiation; 1 h and 6 h post irradiation, miRNA expression levels were assessed and presented as fold change as compared to non-irradiated controls (0 Gy). Data are expressed as mean ± SEM (*n* = 3), and statistically significant differences are denoted by * *p* < 0.05. The human U6 gene was used as a normalization factor. (**C**) Impact of radiation-induced changes in Let-7 miRNA expression. U-251 cells were collected at 1 h and 6 h post 6 Gy irradiation for RIP assay. RIP assays with human monoclonal antibody anti-EIF2C2 (AGO2) and mouse IgG2a (negative control). miRNA levels in immunoprecipitated samples were determined by qRT-PCR and are reported as percentage with respect to the input sample (%input). Data are expressed as mean ± SEM (*n* = 3), and statistically significant differences are denoted by * *p* < 0.05.

To determine the relative expression of the Let-7 family in U-251 cells under basal conditions, total cellular RNA was collected, and miRNA sequencing(miR-seq) was performed. As shown in Figure 1A, 6 of the top 10 expressed miRNAs in U251 cells were members of the Let-7 family. To investigate the effects of radiation on Let-7 expression, we focused on Let-7a, Let-7b, and Let-7f as representative examples. For this initial analysis, the standard RT-qPCR approach was used to evaluate total cellular miRNA levels. Specifically, U-251 cells were irradiated (6 Gy) and collected 1 and 6 h later, total RNA was isolated, and miRNA levels were quantified.

As shown in Figure 1B, at 1 h after irradiation, there was no significant change in the expression of Let-7a and Let-7b, while a two-fold increase in Let-7f levels was detected. By 6h, all three Let-7 miRNAs had returned to control levels following irradiation. In addition to changes in the cellular transcriptome, the effects of radiation on functional Let-7 miRNAs were defined using the AGO2-RIP assay to measure the level of Let-7 miRNA bound to AGO2 (Figure 1C). For these experiments, U251 cells were irradiated (6 Gy); 1 and 6 h later, they were subjected to immunoprecipitation using anti-AGO2 antibody or IgG (negative control), followed by RNA extraction and quantitation of Let-7a, Let-7b, and Let-7f. Data for the AGO2 and IgG groups were expressed as % input miRNA (Figure S1). At 1 h and 6 h after irradiation, there was dramatic decrease in Let-7a, Let-7b, and Let-7f miRNAs bound to AGO2 as compared to non-irradiated controls (Figure 1C). No significant changes in the IgG samples were detected, confirming the specificity of AGO2-Let-7 interactions. These results suggest that, in contrast to Let-7 levels as measured in the transcriptome, radiation induces a rapid and sustained reduction in the levels of functional Let-7a, Let-7b, and Let-7f.

### 2.2. Modifying Let-7 Expression and U-251 Cell Radiosensitivity

**Figure 2:**
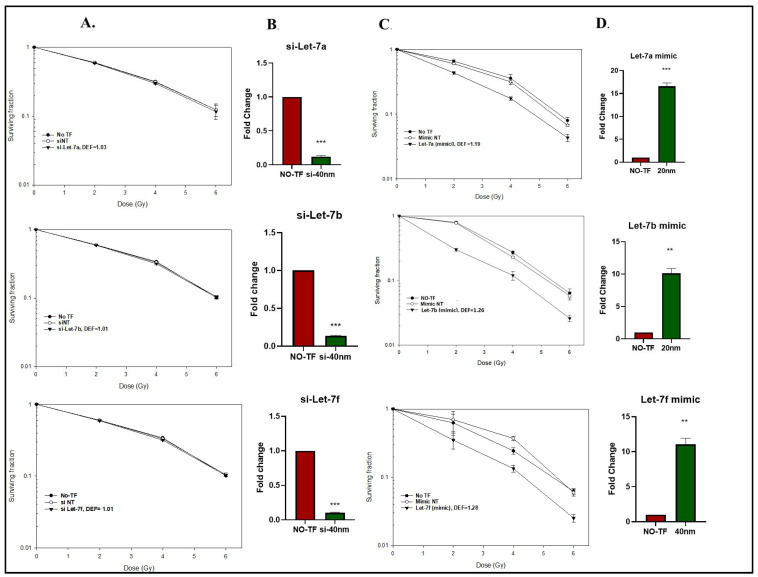
Impact of radiation-induced changes in Let-7 miRNA expression. U-251 cells were collected at 1 h and 6 h post 6 Gy irradiation for RIP assay. (**A**,**C**) Clonogenic survival analysis of U-251 cells after radiation and transfected with Let-7 inhibitors, mimics, or non-target (NT) controls. Cells were seeded at clonal density 48 h post-transfection, and colony formation was evaluated after 2 weeks. Dose enhancement factors (DEFs) were determined at a surviving fraction of 0.1. Data are expressed as mean ± SEM (*n* = 3). (**B**,**D**) Let-7 miRNA expression after mimic or inhibitor transfection. U-251 cells were transfected with inhibitors and mimics for Let-7a, 7b, and Let-7f, and miRNA levels were measured 48 h post-transfection. Data are normalized to U6 and expressed as fold change relative to non-transfected cells (NO-TF) cells. Data are expressed as mean ± SEM (*n* = 3), and statistically significance is denoted as: ** *p* < 0.01, *** *p* < 0.001.

Aiming towards defining the cellular consequences of the radiation-induced decrease in Let-7-AGO2 binding, we performed clonogenic assays to determine the radiosensitivity of U-251 cells after knockdown (siRNA) or enhanced expression (mimic) of Let-7a, Let-7b, or Let-7f. Specifically, 48 h after transfection with siRNA or mimic, cells were trypsinized, seeded at clonal density, and irradiated 16 h later. As shown in Figure 2A, siRNA-mediated knockdown of these Let-7 miRNAs had no effect on radiation-induced cell death. The decrease in the Let-7 miRNAs after siRNA transfection was verified by RT-qPCR (Figure 2B). The knockdown alone had no effect on cell survival of non-irradiated cells with surviving fractions after Let-7a, Let-7b, and Let-7f knockdown of 1.01 ± 0.003, 1.02 ± 0.007, and 0.93 ± 0.08, respectively. In contrast to the siRNA-mediated knockdown, the Let-7 mimics significantly enhanced U251 radiosensitivity with dose enhancement factors (DEFs at 0.1 surviving fraction) ranging from 1.2 to 1.3 (Figure 2C). The increase in the Let-7 miRNAs after mimic transfection was verified by RT-PCR (Figure 2D). The Let-7 mimics alone had no effect on cell survival of non-irradiated cells with surviving fractions after Let-7a, Let-7b, and Let-7f mimics of 1.0 ± 0.01, 1.03 ± 0.03, and 1.01 ± 0.004, respectively. These findings indicate that overexpressing Let-7 miRNA enhances the radiosensitivity of U-251 cells.

### 2.3. Mechanisms Mediating Radio Sensitization Induced by Let-7a Mimic

**Figure 3:**
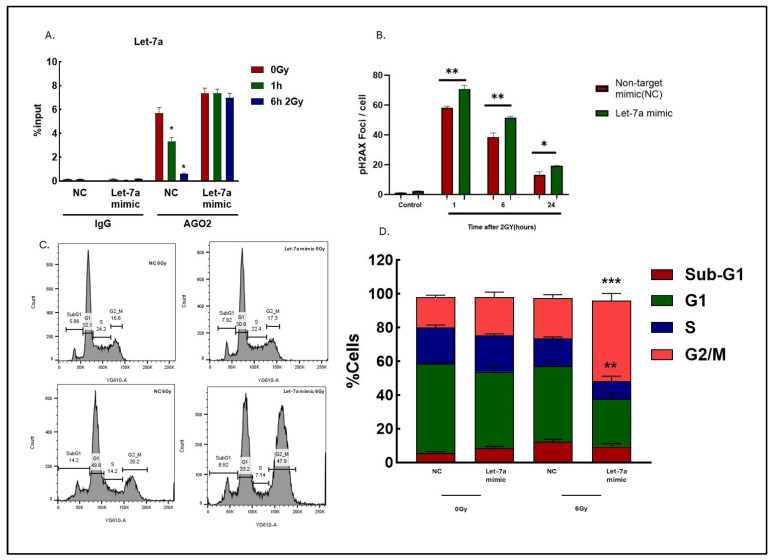
(**A**) Knockdown RIP assays with human monoclonal antibody anti-EIF2C2 (AGO2) and mouse IgG2a (negative control). miRNA levels in immunoprecipitated samples were determined by qRT-PCR and reported as percentage with respect to the input sample (%input). Data are expressed as mean ± SEM (*n* = 3), and statistically significant differences are denoted by * *p* < 0.05. (**B**) γH2AX foci analysis in U-251 cells. Cells transfected with Let-7a mimic or non-target control (NC) for 48 h and irradiated with 2 Gy the following day and collected at specified time points for foci analysis. Data are expressed as mean ± SEM from three independent experiments, and statistically significant differences are denoted as: * *p* < 0.05, ** *p* < 0.01. (**C**) Cell cycle analysis was carried out using flow cytometry in U-251 cells. Cells were transfected with Let-7a mimic or non-target control (NC) for 48 h, followed by irradiation at 6 Gy and collection after 24 h. Representative flow cytometry histograms of irradiated and non-irradiated Let-7a mimic or non-target control samples. (**D**) Percentages of cells in G0/G1, S, and G2/M phases (** *p* < 0.01, *** *p* < 0.001) vs. Let-7a mimic (0 Gy).

For these studies, Let-7a was used as a representative of the Let-7 family members shown in Figure 1 and Figure 2. The data presented in Figure 2 show that the mimic increased overall expression of Let-7a miRNA. However, this approach does not provide information regarding the levels of functional Let-7a (i.e., Let-7a-AGO2 binding). Thus, the AGO2-RIP assay was used to investigate the effects of the mimic on Let-7a-AGO2 binding after irradiation (Figure 3A). Irradiation of cells transfected with non-targeted mimic control (NC) resulted in a significant decrease in Let-7a-AGO2 binding, consistent with data shown in Figure 1C. However, in cells transfected with the mimic, the overexpression of Let-7a (see Figure 2D) prevented the radiation-induced decrease in Let-7a-AGO2 binding, indicating the maintenance of functional Let-7a. To further investigate the consequences of the maintenance of Let-7a after irradiation, components of the DNA damage response (DDR) were evaluated. Initial studies addressed γH2AX foci, which correspond to DNA double strand breaks (DSBs), the lethal event mediating radiation-induced cell death. U-251 cells were transfected with the Let-7a mimic for 48 h, irradiated (2 Gy), and collected for foci quantitation at various time points up to 24 h. In the NC cells, the number of γH2AX foci was highest at 1 h and then declined over to 24 h period, consistent with the repair of radiation-induced DSBs. Regarding the treated mimic, γH2AX foci in unirradiated cells were the same as in the NC cells, indicating that exposure to the mimic alone did not induce DSBs. However, at each point after irradiation, the number of foci was significantly greater in comparison to NC-treated cells (Figure 3B) (Figure S2). These data suggest that the mimic and restoration of Let-7a-AGO2 binding after irradiation inhibited the repair of radiation-induced DSBs.

Activation of cell cycle checkpoints also plays a crucial role in the radiation-induced DDR. To investigate the impact of Let-7a on cell cycle distribution following irradiation (6 Gy), flow cytometric analysis was performed on U251 cells 48 h after transfection with the Let-7a mimic or non-target mimic control (NC). Representative histograms are shown in Figure 3C, with data summarized in Figure 3D. In unirradiated cells, Let-7a mimic alone had no effect on cell cycle phase distribution. However, following irradiation, in the Let-7a mimic-expressing cells, there was a significant increase in the percentage of cells in G2/M, along with a reduction in the S-phase fraction, as compared to the irradiated NC transfected cells. The results shown in Figure 2 and Figure 3B,C suggest that preventing the radiation-induced reduction in Let-7a-AGO2 binding enhances U-251 radiosensitivity, at least in part, by inhibiting the critical components of the DNA damage response.

**Figure 4:**
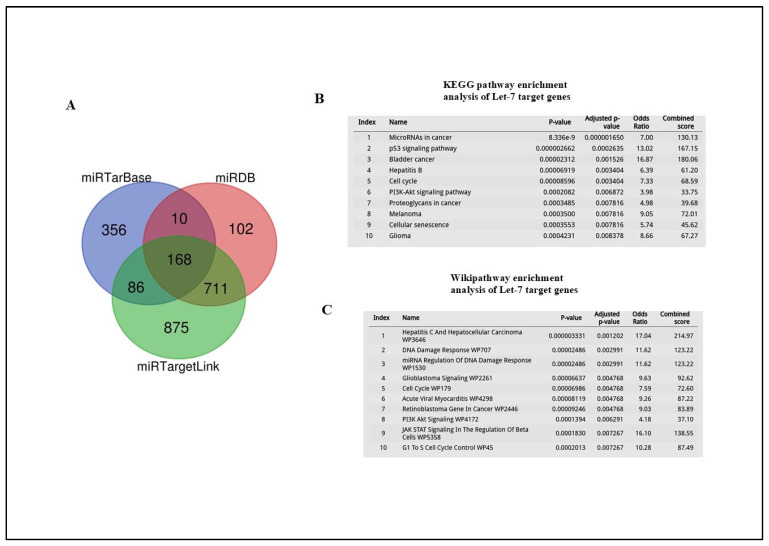
(**A**) miRNA target prediction. The Venn diagram illustrates the number of targets of let-7 miRNAs predicted by each tool. (**B**) KEGG pathway enrichment analysis of Let-7 target genes showing top 10 highly most enriched pathways with significance (*p* < 0.001), carried out using the Enrichr tool. (**C**) Wikipathway enrichment analysis of Let-7 target genes, carried out using the Enrichr tool (*p* < 0.001).

We then determined whether the compromised DDR after irradiation was consistent with the mRNA targets of Let-7 miRNA. To this end, we integrated results from three prediction algorithms: miRTarBase (https://mirtarbase.cuhk.edu.cn/, accessed on 13 January 2025), miRTargetLink2.0 (https://www.ccb.uni-saarland.de/mirtargetlink2, accessed on 13 January 2025), and miRDB (http://mirdb.org/, accessed on 13 January 2025). As shown in Figure 4A, there was an overlap of 168 Let-7 target genes identified by three databases, and these were subsequently used for gene enrichment analysis. The KEGG and Wiki pathway enrichment analysis indicated that Let-7 miRNAs target genes were involved in DDR-related processes such as the cell cycle and DNA damage response (Figure 3C and Figure 4B).

### 2.4. Effects of Radiation on Let-7-AGO2 Binding in Other Tumor and Normal Cell Lines

**Figure 5:**
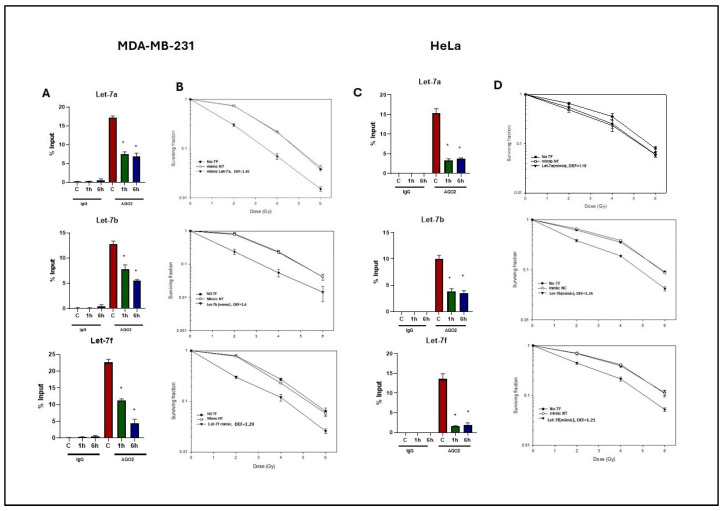
Effect of radiation on Let-7 miRNA binding to AGO2 in tumor cell lines (MDA-MB-231 and HeLa) 1 h and 6 h post 6 Gy irradiation. (**A**,**C**) RIP experiments with anti-AGO2 antibody (or IgG as control) on cellular lysates of MDA-MB-231 and HeLa, respectively. The Let-7 miRNA expression levels in immunoprecipitated (IP) samples were calculated using qRT-PCR and are reported as a percentage relative to the input sample. Data are expressed as mean ± SEM (*n* = 3), and statistically significant differences are denoted by * *p* < 0.05. (**B**,**D**) Clonogenic survival analysis of MDA-MB-231 and HeLA cells post-transfection with Let-7a miRNAs for 48 h and radiation exposure of 6 Gy. Dose enhancement factors (DEFs) were determined at a surviving fraction of 0.1. Data are expressed as mean ± SEM (*n* = 3).

To determine whether the radiation-induced effects on Let-7-AGO2 binding were unique to U-251 glioma cells, studies were extended to MDA-MB-231(breast cancer) and HeLa (cervical cancer) cell lines. RIP analysis was performed to analyze the Let-7-AGO2 binding, as described in Figure 2. For both tumor cell lines, AGO-2 binding to Let-7a, Let-7b, and Let-7f was significantly reduced at 1 and 6 h after irradiation (Figure 5A,C), similar to U-251 (Figure 2). To determine the cellular consequences of the radiation-induced decrease in Let-7-AGO2 binding in MDA-MB-231 and HeLa cell lines, the clonogenic survival assay was used to identify changes in radiosensitivity. As for the U-251 cells, the Let-7 mimics enhanced MDA-MB-231 and HeLa cell radiosensitivity, as determined by clonogenic analysis (Figure 5B,C). For both tumor cell lines, Let-7a, Let-7b, and Let-7f mimics resulted in an increase in radiosensitivity with DEFs of 1.3–1.6 for MDA-MB-231 and 1.2 for HeLa. The Let 7 mimics alone had no effect on the survival of non-irradiated MDA-231 cells, with surviving fractions after Let-7a, Let-7b, and Let-7f mimics of 0.95 ± 0.05, 1.11 ± 0.06, and 1.07 ± 0.05, respectively. Similar results were obtained for HeLa cells with surviving fractions after Let-7a, Let-7b, and Let-7f mimics of 0.81 ± 0.02, 0.92 ± 0.10, and 0.96 ± 0.02, respectively.

**Figure 6:**
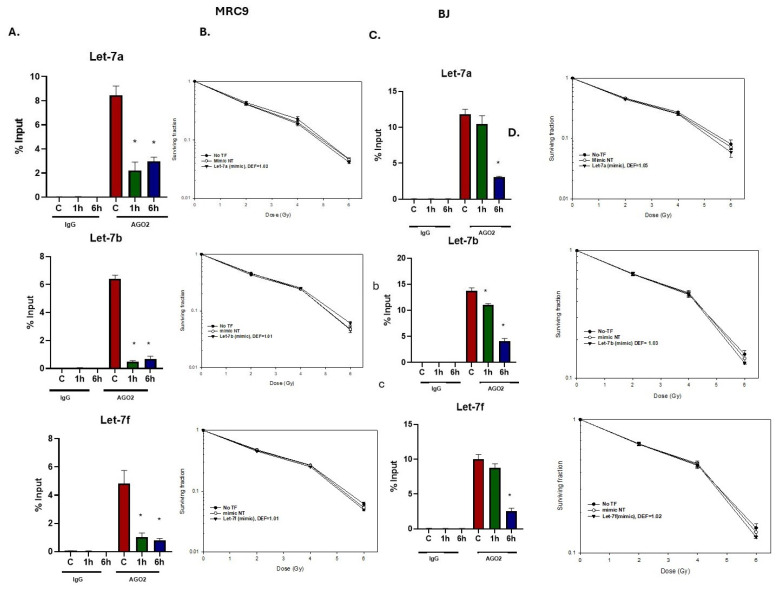
Influence of radiation on Let-7 miRNA binding to AGO2 in normal fibroblast cell lines (MRC9 and BJ) 1 h and 6 h post 6 Gy irradiation. (**A**,**C**) RIP assay was performed using anti-AGO2 antibody (or IgG as control) on cellular lysates of MRC9 and BJ, respectively. The Let-7 miRNA expression levels in IP samples were measured using qRT-PCR and expressed as percentage relative to the input sample. Data are expressed as mean ± SEM (*n* = 3), and statistically significant differences are denoted by * *p* < 0.05. (**B**,**D**) Clonogenic survival analysis of MRC9 and BJ cells post-transfection with specified Let-7 miRNAs for 48 h and radiation exposure of 6 Gy. DEFs were determined at a surviving fraction of 0.1. Values are presented as mean ± SEM from three independent experiments.

Extension of this analysis to the normal fibroblast cell lines MRC9 and BJ cells also showed that radiation reduced AGO2 binding to Let-7a, Let-7b, and Let-7f, consistent with the tumor cell lines (Figure 6A), although in BJ cells, the reduction occurred primarily at 6 h after irradiation (Figure 6C). However, the Let-7a, 7b, and 7f mimics had no effect on radiosensitivity of both normal cell lines (Figure 6B,D). The Let 7 mimics alone had no effect on the survival of non-irradiated MRC9 cells, with surviving fractions after the transfection of Let-7a, Let-7b, and Let-7f mimics of 1.10 ± 0.05, 1.21 ± 0.12, and 1.15 ± 0.07, respectively. Similar results were obtained for BJ cells with surviving fractions after the transfection of Let-7a, Let-7b, and Let-7f mimics of 1.15 ± 0.03, 1.01 ± 0.01, and 1.13 ± 0.06, respectively. These results suggest differences between normal and tumor cell lines regarding the role of the Let-7 family as a determinant of radiosensitivity.

## 3. Discussion

Radiation-induced gene expression has long been considered an adaptive or protective response against radiation-induced injury or death. Analogous to prokaryotic cells, the process in eukaryotes was initially assumed to be mediated via changes in gene transcription. Arguing against this scenario is the poor correlation between IR-induced changes in mRNAs and their corresponding proteins [16]. Subsequent studies comparing changes in total mRNA to those in polysome-bound mRNA after irradiation indicated that radiation-induced gene expression is mediated primarily via translational control [17]. However, the mechanisms mediating the radiation-induced translational control of gene expression have not been defined. Towards this end, in the study described here, we focused on miRNA, specifically on the AGO2/miRNA interaction, using Let-7 as a representative miRNA.

AGO2, a critical component of the RNA-induced silencing complex, is essential for mediating post-transcriptional gene suppression [18,19]. Changes in miRNA availability or efficient RISC loading due to radiation exposure modulates targeting of mRNAs involved in DNA repair, cell cycle regulation, and apoptosis [20,21]. The data presented show that radiation reduces the association of a major family of miRNAs (Let-7) and AGO2 in the absence of significant changes in transcriptome. These results imply that radiation reduces the level of functionally active Let-7, allowing for an increase in the expression of target genes.

Recent studies of AGO2-miRNA have shown that inhibition of translation initiation is an early step in miRNA-mediated gene silencing occurring before and independently of mRNA degradation [22,23,24], consistent with the concept that changes in miRNA binding to AGO2 cause rapid changes in the gene expression. As shown, radiation-induced changes in AGO2-miRNA were detected at 1 h, suggesting the potential to mediate a rapid change in radiation-induced translational control of gene expression.

Investigating the functional significance of the radiation-induced decrease in the AGO2-Let-7 binding revealed that preventing the reduction in AGO2 bound Let-7 miRNAs using Let-7a mimics increased tumor cell radiosensitivity. These results imply that the reduction in functional Let-7 plays a fundamental role in protecting tumor cells from radiation-induced death. More specifically, these data suggest that the rapid reduction in AGO2-Let-7 binding allows for the expression of Let-7 target genes that participate in cell cycle regulation and DNA repair, as shown in Figure 3. Although we did not directly measure these targets in the current study, future studies employing transcriptomic or protein-level analyses will be critical to validate their role in observed radiosensitivity.

In contrast, normal human fibroblasts showed reduced AGO2/Let-7 binding after radiation but no effect on radiosensitivity upon Let-7 overexpression, suggesting a fundamental difference between tumor and normal cells in the mechanisms mediating radiation-induced translational control of gene expression. Together, these findings highlight that radiation influences the post-transcriptional regulation of miRNAs through reducing binding to AGO2. These findings indicate regulation of miRNA-AGO2 interaction as a novel mechanism that drives rapid translational activation of gene expression in response to radiation.

## 4. Methods

### 4.1. Cell Culture

The U-251 Glioblastoma (GBM) cell line was obtained from the Division of Cancer Treatment and Diagnosis Tumor Repository (DCTD) at the National Cancer Institute (NCI). MRC9 (normal lung fibroblasts), BJ (normal human fibroblast cell line), MDA-MD-231(Breast adenocarcinoma), and HeLa (Cervical Cancer) cells were sourced from the American Type Culture Collection (ATCC, Manassas, VA, USA). U-251, HeLa, and MDA-MB-231 cells were cultured in DMEM supplemented with 10% FBS (Invitrogen, Waltham, MA, USA). MRC9 and BJ cells were grown in MEM supplemented with 10% FBS, L-glutamine, and sodium pyruvate (Invitrogen, Walthman, MA, USA). All cells were maintained at 37 °C in an atmosphere of 5% CO_2_ and 95% relative humidity. Cells were irradiated using a 320 kV X-ray source (Precision X-ray, Inc., Madison, WI, USA) equipped with a 2.0 aluminum filter, operating at 300 kVp and 10 mA, delivering a dose rate of 2 Gy/min.

### 4.2. miRNA Sequencing

miRNA sequencing was performed on NextSeq 2000 P2 using the QIASeq^®^ miRNA UDI Library Kit with single-end sequencing. Raw base calls, generated as the primary sequencing output, were converted into FASTQ input files using Bcl2fastq V2. 20 software, which automatically separates multiplexed samples. Adaptor sequences were trimmed from the reads before alignment using Trimmomatic 0.34 [25]. Reads with length greater than 27 bases were discarded for miR-seq analysis using miRDeep2 [26] with the human reference genome hg38. Bowtie 1. 2.2. aligner [27] was used for mapping, and the average mapping rate across all samples was 92%. miRDeep2 identifies novel miRNAs through stringent read length filtering for downstream analysis. Data processing was performed using the R software(version 4.3.1) and the package (version 2.0.0). Raw count data for mature miRNAs was imported into edgeR [28] and normalized using “upper quartile”, suggested for miRNA data. The data discussed in this publication have been deposited in NCBI’s gene expression omnibus (GEO) and are accessible through GEO Series accession number (GSE290025).

### 4.3. Total RNA Isolation and Quantitative Real-Time PCR (qRT-PCR)

Total RNA was isolated from U-251 cells using the RNeasy mini kit (Qiagen, Hilden, Germany) according to the manufacturer’s recommendations. The RNA concentration was determined using a NanoDrop^TM^ 2000 spectrophotometer (Thermo Fisher Scientific, Rockford, IL, USA). Subsequently, the total RNA was reverse-transcribed into cDNA using the miRCury LNA RT kit (Qiagen, Venlo, The Netherlands), which enables rapid miRNA polyadenylation, coupled with reverse transcription in a single step. Real-time quantitative PCR was performed using the miRCury LNA SYBER Green PCR kit (Qiagen, Hilden, Germany) and ABI 7500 FAST Real-Time PCR instrument (Applied Biosystems, Foster City, CA, USA). The reaction conditions included pre-denaturation at 95 °C for 30 s, denaturation at 95 °C or 5 s, annealing/extension at 56 °C for 60 s, and a total of 40 cycles. The relative expression of Let-7 miRs was evaluated using the 2-delta delta Ct method. U6 served as the endogenous control for all miRNAs.

### 4.4. siRNA and miRNA Mimic Transfection

Transfection of miRNA mimics and inhibitors was carried out in opti-MEM using Lipofectamine RNAiMax as per the manufacturer’s instructions. The sequences of hsa-Let-7a-5p, hsa-Let-7b-5p, and hsa-Let-7f-5p (miRCury LNA miRNA mimics) and their knockdown sequences (miRCury LNA miRNA Inhibitor), locked nucleic acid (LNA) (Qiagen, Hilden, Germany), were transfected for 48 h to induce overexpression and knockdown of Let-7 miRNAs. The Negative miRCURY LNA miRNA mimic control and miRCURY LNA miRNA Inhibitor control were used as negative controls.

### 4.5. AGO2-RNA Immunoprecipitation Assay (RIP)

The AGO2-RIP assay was carried out to determine interactions between AGO2 and the Let-7 family of miRNAs, as described previously [29,30]. AGO2 protein-bound miRNA IP was conducted using the Ribocluster Profiler Rip Assay kit protocol (MBL international Corporation, Woburn, Woburn, MA, USA) according to the manufacturer’s instructions. Protein complexes were immunoprecipitated with an anti-EIF2C2 (AGO2) antibody (Code no. RN003M); mouse IgG isotype control (Code no. M075-3) was used as a negative control (MBL International, Woburn, MA, USA). miRNA lysis buffer plus dithiothreitol (DTT; Invitrogen, Waltham, MA, USA), provided by the manufacturer, was used to lyse cells, and protein G agarose beads (Thermo Fischer Scientific, Waltham, MA, USA) were used to preclear lysates in DTT-miRNA wash buffer supplied by the manufacturer. The precleared lysates were transferred to tubes containing anti-AGO2 or IgG-immobilized agarose beads. After overnight incubation, the RNA/protein complexes bound to antibody-immobilized beads were separated to extract AGO2-bound RNA and proteins. Approximately 40 mg protein lysates and 10% input from 2 × 10^7^ cells were used for RNA isolation and Western Blotting (WB), respectively. Additionally, 90% of final beads were used for RNA extraction, and 10% for WB. AGO2-bound RNAs (RIP RNA) and input RNA were extracted and purified with reagents provided in the kit, according to the manufacturer’s instructions. Isolated input RNA (100 ng) and RIP RNA were subjected to cDNA conversion using the miRCury LNA RT kit (Qiagen, Venlo, The Netherlands) and analyzed by qPCR. Enrichment was calculated using the percent input normalization method [31].

### 4.6. Clonogenic Survival

To assess radiosensitivity, cells were transfected with Let-7 miRNA mimics or inhibitors (Qiagen, Hilden, Germany), as well as their respective negative controls. Also, 48 h post-transfection, cells were replated in 6-well plates at clonal density and irradiated after 16 h. Cells were cultured for approximately 2 weeks to allow for colony formation. Colonies were stained with 0.5% crystal violet (Sigma-Aldrich, Fukushima, Japan) in methanol. The number of colonies with ≥50 cells and the surviving fractions were measured. Radiation survival curves were plotted after data normalization to the cytotoxic effects caused by Let-7 miR knockdown or overexpression.

### 4.7. γH2AX Foci

Cells were transfected with Let-7a miRNA mimic or non-target controls (NCs) and plated on chamber slides 48 h post-transfection. At different time intervals after irradiation (1, 6 and 24 h), slides were fixed with 4% formaldehyde, permeabilized with 0.1% Triton X-100 (Sigma Aldrich, St. Louis, MO, USA) and blocked in 10% goat serum in PBS. Primary antibody against phospho-H2AX (Millipore, Burlington, MA, USA) was used, followed by secondary antibody incubation with anti-mouse Alexa Fluor 488(Invitrogen, Waltham, MA, USA). Images of the cells were captured on a Zeiss Axio imager 2 (Carl Zeiss, Oberkochen, Germany) with a 63x oil immersion objective.

### 4.8. Flow Cytometry

For cell cycle analysis, cells were transfected with Let-7a mimic or non-target control mimics (NCs). Forty-eight hours post-transfection, cells were irradiated, and after 24 h, they were fixed in prechilled 70% ethanol and kept overnight at −20 °C. Cells were washed with PBS and stained with 10 µg/mL propidium iodide (PI) containing RNAease (100 µg/mL; Fermentas, Thermoscientific Fischer, Walthman, MA, USA; catalog no.EN0531) at room temperature. Flow cytometry (Fortessa BD bioscience, San Jose, CA, USA) was performed, and at least 20,000 gated events were acquired for each sample. The FL2-A channel (PI) was used to measure fluorescence intensity. The percentage of cells in the G0/G1, S, and G2/M phases under specified conditions was analyzed using FlowJo software (FlowJo LLC, Ashland, OR, USA, version 2023).

### 4.9. miRNA Target Prediction Databases and Pathway Enrichment Analysis

The freely available online databases miRDB [32], miRTarBase [33], and miRTargetLink 2.0 [34] were used for the prediction of miRNA targets. mirDB database uses computational algorithm miRTarget to predict miRNA binding sites in the seed sequence of the 3′UTR region of target mRNA. miRTarBase considers experimentally validated miRNA target interactions (MTIs), and miRTargetLink identifies target mRNA by complementary base pairing between miRNAs and target genes within the 3′UTR region of mRNA. Common target genes predicted by all three databases were selected for further analysis. KEGG and WikiPathways analysis were performed for predicted target genes using Enrichr (a gene set enrichment analysis tool) [35]. Pathways with the highest number of genes and a *p*-value < 0.001 were considered significant.

### 4.10. Statistical Analysis

Statistical significance was evaluated by Student’s *t*-test and ANOVA using Graph Pad Prism 10 software.

## Data Availability

The miRNA expression data generated in this study are publicly available in the Gene expression omnibus (GEO) GSE290025 (https://www.ncbi.nlm.nih.gov/geo/query/acc.cgi?acc=GSE290025, accessed on 1 June 2025). All other data is included in this study.

## Supplementary Materials

The following supporting information can be downloaded at: https://www.mdpi.com/article/10.3390/ijms26178483/s1.
