# Supplementary material for: Radiation Modifies Let-7 miRNA Binding to AGO2 Independent of Changes in Transcription to Influence Tumor Cell Radiosensitivity
Source: Int J Mol Sci. 2025 Sep 1;26(17):8483. doi: 10.3390/ijms26178483 (PMC12429687; doi:10.3390/ijms26178483)
Supplement: Supplementary file 1 [file ijms-26-08483-s001.zip › ijms-3804580-supplementary.pdf]

Figure S1: Representative Western blot analysis of RIP assays with human monoclonal antibody anti-EIF2C2 (AGO2) and mouse IgG2a (isotype control)

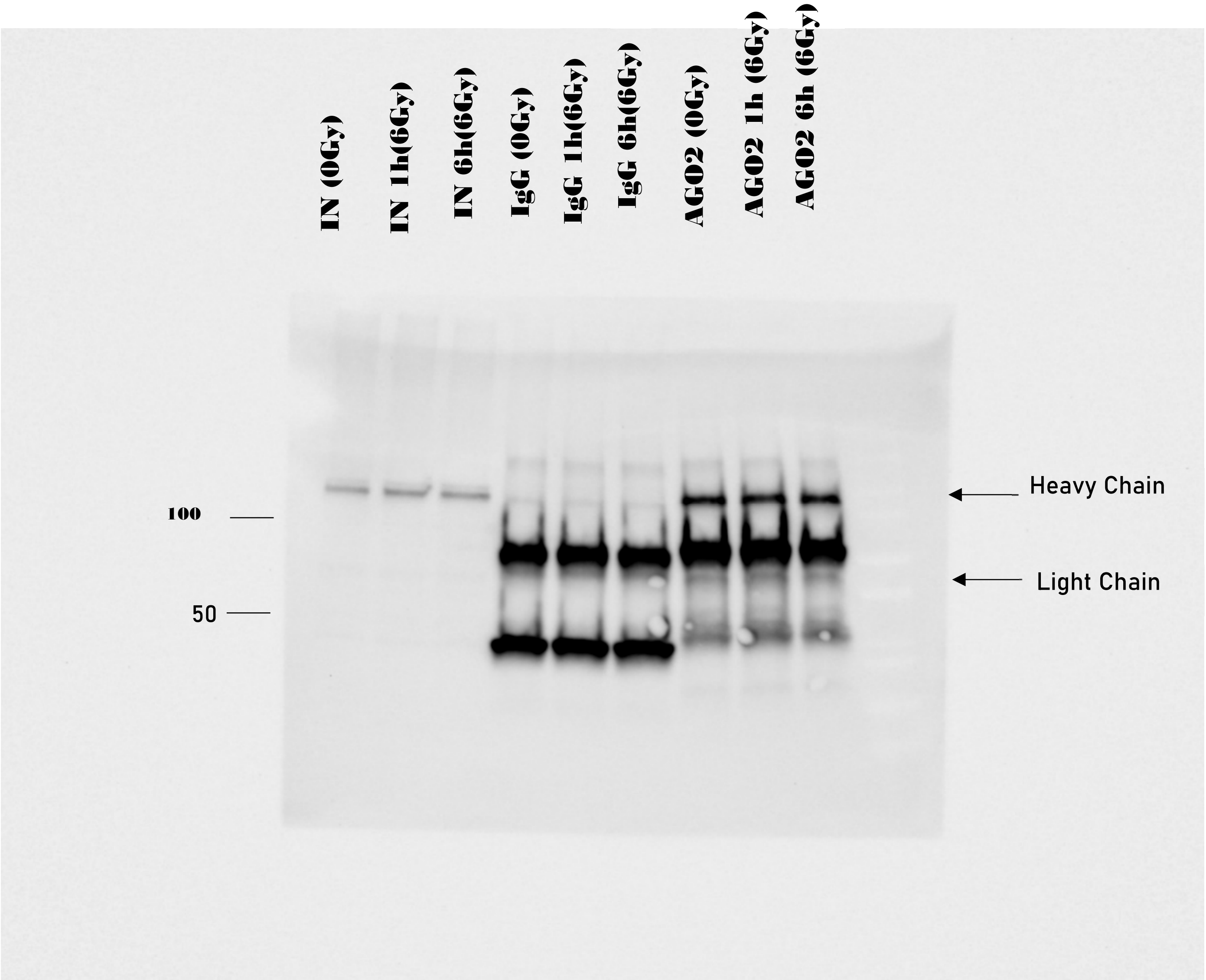

Figure S1: RIP assays were performed using human monoclonal antibody anti-EIF2C2 (AGO2) or mouse IgG2a (isotype control). Western Blot analysis shows immunoprecipitated proteins with anti-AGO2 (IP). IgG2a are the negative controls. IN(Input) made up 10 % of the cytoplasmic lysate used for each IP sample.

Figure S2: Representative fluorescence images of γH2AX foci in U-251 cells

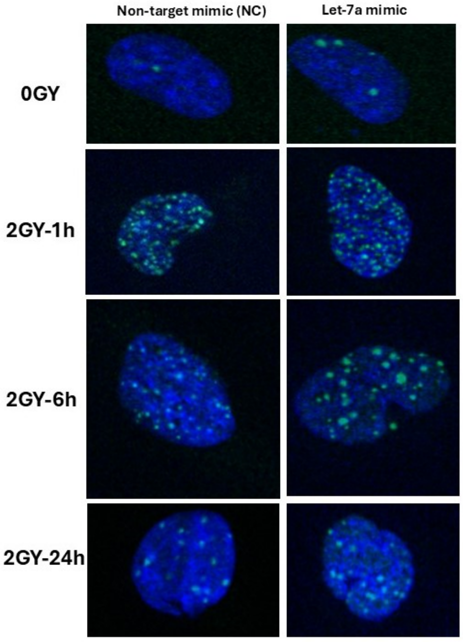

Figure S2: γH2AX foci analysis in U-251 cells; Cells were treated with Let-7a mimic or non-target mimic control(NC) for 48 hours and irradiated (2GY) the following day and collected and fixed at specified time points(1, 6 and 24h) for foci analysis using fluorescence microscopy
